# Rapid autofluorescence based 3D optical imaging of the pancreatic cancer milieu at mesoscopic scale – stain-free volumetric segmentation

**DOI:** 10.1038/s41598-026-54433-z

**Published:** 2026-05-31

**Authors:** Joakim Lehrstrand, Tomas Alanentalo, Martin Isaksson Mettävainio, Sara Jacobson, Asif Halimi, Ulf Ahlgren, Oskar Franklin

**Affiliations:** 1 Dept. of Medical and Translational Biology, Umeå University, Umeå, Sweden; 2 Dept. of Medical Biosciences, Umeå University, Umeå, Sweden; 3 Dept. of Diagnostics and Intervention, Surgery, Umeå University, Umeå, Sweden

**Keywords:** Biological techniques, Cancer, Oncology

## Abstract

Although major advances have been made in the field of mesoscopic imaging and associated tissue clearing protocols, these applications are greatly challenged when applied to imaging of pancreatic ductal adenocarcinoma (PDAC) tissue. Most importantly, penetration of labelling agents, typically antibodies, can be drastically reduced from the characteristically dense PDAC stroma. To circumvent this issue, we present a method by which machine learning assisted segmentation is applied to resolve the 3D PDAC microarchitecture from autofluorescence (AF) based light-sheet fluorescence microscopy (LSFM) scans. Hereby, PDAC tissue features could be studied in 3D space without the need for labelling or sectioning. In this proof of principle study, we applied this imaging pipeline on surgical specimens from five PDAC patients and normal pancreatic tissue, generating mosaics of cm^3^-sized tissue discs at micrometre resolution, each on scanning depths corresponding to thousands of standard pathological 2D tissue sections. Using this method, we generated 3D volumes for quantification of blood vasculature, neoplastic epithelium, islets of Langerhans and stromal components. We further showcase the potential for downstream 2D histochemical and immunohistochemical analysis of scanned specimen. As such, the method may facilitate studies of metastatic routes, vessel microarchitecture, islet phenotypes, and spatial relationships in the PDAC tumour microenvironment.

**Supplementary Information:**

The online version contains supplementary material available at 10.1038/s41598-026-54433-z.

## Introduction

Histopathology for research and clinical diagnostics is dominated by histology, histochemistry and antibody staining of thin tissue sections in 2D. While imaging in 2D is cheap and high throughput, it may obscure and limit assessments of spatial relationships in the tumour microenvironment that would need high resolution imaging in 3D to be unravelled. Recent advances in optical imaging techniques have provided tools for 3D visualization of cancer tissue^1–3^, but their applications are hampered by poor antibody penetration, limited size of samples or tissue destruction. Pancreatic ductal adenocarcinoma (PDAC) presents specific challenges, as its tumour microenvironment is characterized by a dense desmoplastic tumour stroma^4^ that can drastically impede antibody penetration. Given that the PDAC microenvironment may influence cancer dissemination, chemotherapy resistance, vessel microanatomy and Langerhans islet function, techniques to characterize its structure with maintained spatial context are highly warranted.

Light sheet fluorescence microscopy (LSFM), also referred to as selective plane illumination microscopy (SPIM)^5–8^, is based on the perpendicular laser illumination of the field of view with a thin slice at high optical resolution. The method is comparable to confocal microscopy in generating high resolution optical sections but generates images at a faster speed and greater depth, with low levels of photodamage/bleaching. LSFM is therefore commonly applied to generate 3D images of large, cleared tissue specimens. We have previously employed this technique to study the innate autofluorescent (AF) properties of PDAC tissue for islet visualization and how hypointense regions allow for delineating the tumour border^1^.

In this study we utilize LSFM to generate high-resolution cm^3^-sized 3D scans of cleared tissue discs from PDAC surgical specimens. We further showcase how machine-learning assisted annotation of the inherent AF properties can be used to generate volumetric 3D renderings of neoplastic epithelium, blood vasculature, periductal stroma and pancreatic islets. Finally, we demonstrate that routine Haematoxylin & Eosin (HTEX) and antibody labelling can be readily performed on 2D sections of the 3D scanned samples. This in-depth spatial characterization of the PDAC microenvironment can provide useful information on metastatic routes, chemotherapy deliverance, growth patterns and islet morphology. In a clinical setting, the method has potential applications to identify tumour borders for radicality assessment and selection of areas for classification, especially since routine histopathology is not hampered by the preceding 3D imaging.

## Materials and methods

### Ethics approval and consent to participate

This study was conducted according to the principles of the Declaration of Helsinki and was approved by the Swedish National Review Authority (DNR: 2016/384 − 31 and 2019–04593). The patients have provided consent for tissue collection.

### Tissue preprocessing and clearing

PDAC surgical specimens were fixed in formalin, retrieved, and washed in 96% (v/v) ethanol to remove excess fat in the tissue, until the washes become transparent. The samples were then stored in 96% (v/v) ethanol at 4 °C until use. Preparation for optical clearing was performed essentially as described^1^. In brief, the specimens were acclimated to 100% MeOH and subjected to freeze-thaw cycles to enhance solvent penetration, from RT to −80 °C and back, 1 h for each for step. To reduce pigmentation and innate AF, the samples were kept in bleaching solution, (H_2_O_2_; Cat. No. H1009; Sigma-Aldrich, Merck) at a final concentration of 15% (v/v)), dimethyl sulfoxide (DMSO; Cat. No. D5879; Sigma-Aldrich, Merck) and MeOH (H_2_O_2_:DMSO: MeOH) in a ratio of 3:1:2 for 6 h at RT and, then incubated in fresh bleaching solution overnight. After overnight incubation, the samples were washed twice at RT in 100% MeOH, 30 min per wash. The samples were then cleared in BABB, a 1:2 mixture of benzyl alcohol (Cat. No. 109626; VWR) and benzyl benzoate (Cat. No. 105860010; Acros Organics) solution was replaced every 3 h until the MeOH was washed out and the samples were completely cleared. Dehydration and clearing together results in an estimated tissue shrinkage of ~ 5%^9^.

### Light sheet fluorescence microscopy imaging

LSFM imaging was performed using an UltraMicroscope II (Miltenyi Biotec, Germany), equipped with a 1× Olympus objective (Olympus PLAPO 2XC) coupled to an Olympus MVX10 zoom body, A 3000-step chromatic correction motor and a lens corrected dipping cap MVPLAPO 2× DC DBE objective. Mosaic scans were generated with a 0.63x magnification, whilst selected ROIs were scanned at 1.25x magnification with a numerical aperture of 0.141, at the entire sample depth with a 10–15 μm step size, giving a voxel size of 4.79 × 4.79 × 15 μm for mosaics and 2.42 × 2.42 × 10 μm for ROIs, with a dynamic focus set to 10 images across the field of view. Light sheets were merged using the built-in projection function. Filter sets used were as follows: Ex: 650/45, Em: 750/60 and Ex: 470/40, Em: 525/50, and the exposure time for all channels were kept at 300 ms. The resultant datasets were saved in *.ome.tif format native to ImSpectorPro software (version 7.1.15; LaVision Biotec), before being converted into 3D projection *ims files using Imaris File Converter software (version 10.0.0; Bitplane). The final dimensions of the ROIs for the analyses were 4 × 4.5 × 1.5–3 mm in x, y and z respectively. The resulting *.Ims files ranged between 3 and 8 GB before analysis.

### Image analysis

LABKIT^10^ utilizes a random forest^11^ supervised pixel classifier algorithm developed for large datasets where minimal annotation can result in effectively resolving structures of interest. The mesoscopic image data was manually annotated where several foreground classes were generated (i.e. cancer epithelium, vessels, ductal structures and islets) and used for training. The training of a classifier was conducted by loading the complete image into LABKIT and sparingly annotating (draw) the pixels belonging to structures of interest on a 2D optical sectional plane. In addition, regions of “secondary” structures (such as stroma) were annotated as “background”.,. Pixel classifier filters included in training were as follows; original image, gaussian blur, difference of gaussians, gaussian gradient magnitude, Laplacian of gaussian and hessian eigenvalues all applied for each sigma (1,0; 2,0; 4,0; 8,0). After a first round of training, a resulting segmentation mask based on the first annotation was displayed over the original channel across the entire Z-stack. The segmentation was then manually evaluated across the specimen to determine the accuracy. Then, the structure of interest or background was further annotated to refine the segmentation. Here, annotation of several areas was required, including objects of different sizes and morphology where relevant, and on several Z-depths as this affects the pixel intensity which is the basis for the training and the resulting segmentation. Therefore, several rounds of additional annotation and training were implemented on a per sample basis until the classes were effectively segmented or until background objects were sufficiently separated to allow for manual removal downstream in Imaris. Since there were inherent morphological variation, size and spectral differences between samples and ROIs, this procedure was carefully repeated on each sample to allow for a more accurate segmentation of the tissue features. The volumetric data was then generated in Imaris and statistics for the surfaces were extracted as *.xml.

### **Tissue retrieval and immunohistochemistry**

Samples were washed 3 times for 3 h in 100% MeOH to remove all traces of BABB, followed by stepwise rehydration in a series of 70%, 50%, 30% and 0% (v/v) methanol to 1 × PBS for 30 min per step, at RT with gentle shaking. After rehydration, the tissue slabs were cryoprotected by incubating in 30% (w/v) sucrose in 1 × PBS overnight at 4 °C. The samples were then embedded and snap frozen in NEG-50 (Cat. No. 11912365; Fisher Scientific) and stored at − 80 °C. 10-µm-thick sections were collected onto SuperFrost Plus glass slides (Cat. No. 10149870; Fisher Scientific) and air dried before storage at −20 °C.

For immunofluorescence staining, tissue sections were blocked in 10% fetal bovine serum (FBS; Cat. No. 11550356; Sigma-Aldrich, Merck) for 1 h at RT and stained with rabbit anti-CD31 (diluted 1/500, Cat. No. ab28364; Abcam), mouse anti-CK7 (diluted 1/500, Cat. No. M7018; Dako), rabbit anti-CK19 (diluted 1/500, Cat. No. ab52625; Abcam) and mouse anti-alpha-SMA (Cy3-conjugated, diluted 1:1000, Cat. No. C6198; Sigma-Aldrich) in blocking solution for 1 h at RT. Slides were washed 3 times for 5 min in TBST (0.15 M NaCl, 0.1 M Tris-HCl and 0.1% Triton^®^ X-100) and the following secondary antibodies in blocking solution for 30 min at RT: 647 AffiniPure-F(ab’)₂ Fragment Donkey Anti-Mouse (Cat. No. 715-606-150; Jackson, diluted 1:500) or Donkey anti-Rabbit (Cat. No. 711-606-152; Jackson ImmunoResearch; diluted 1:500) and 594 AffiniPure-F(ab’)₂ Fragment Donkey Anti-Rat (Cat. No. 712-586-153; Jackson ImmunoResearch; diluted 1:500), followed by staining with 4′,6-diamidino-2-phenylindole (DAPI, diluted 1:10000 in PBS) for 5 min at RT. After incubation, slides were washed 3 times for 5 min in TBST and mounted with Vectashield^®^ mounting medium (Cat. No. H-1000; Vector Laboratories).

Sections for HTEX staining were first incubated for 5 min in 1x PBS. Then, the samples were stained using Mayers HTX plus (Cat. No. 01825, HistoLab) for 2 min, followed by Eosin Y aqueous solution (Cat. No. SDHT110216, Merck) for an additional 2 min. The samples were then dehydrated in 95% ethanol for 1 min 2 times and then in 99.5% ethanol for an additional 2 times of 1 min incubation. Finally, the samples were washed in Xylene (Cat. No. 28975.291, VWR) for 5 min before mounting using DPX mounting media (Cat. no. 06522, Sigma-Aldrich) which was left to dry before brightfield imaging.

All 2D sections were scanned using the automated Axio Scan.Z1 Slide Scanner (ZEISS, Germany), equipped with a Colibri 5/7 light source and an Axiocam 506 camera (ZEISS, Germany) with a Plan-Apochromat 20×/0.8 M27 objective and the following filters: DAPI (Ex: 375–395, Em: 455–483), Alexa Fluor 594 (Ex: 574–599, Em: 612–682) and, Alexa Fluor 647 (Ex: 633–647, Em: 635LP). Scans were saved as *.czi files and analysed using ZEN (blue edition) microscopy software (version 3.7.97; ZEISS, Germany).

### Comparative assessment of machine learning-segmentation

To determine the accuracy of the generated machine learning assisted epithelial segmentation, we first matched the original 3D image by carefully aligning optical section (10 μm) planes in Imaris with the cryosections used for immunofluorescence staining. After alignment (best possible match), the segmentation mask was exported as *.tif. Then, a segmentation of the CK19 signal (neoplastic epithelium) was generated in ImageJ first by applying a gaussian filter to remove noise, followed by thresholding. The area fraction of the machine learning segmentation and the immunofluorescent staining was then compared (**Fig. S5**).

### Statistics

Statistical data for volumes generated in Imaris software were exported as Excel (Microsoft Office 365, version 2601) *.xml files. Graphs were generated and statistical tests performed using GraphPad Prism software (version 10.6.1; GraphPad Software, USA). First, the columns (**Fig. 4**) were tested for normality using a Shapiro–Wilk test for normality. Then, the passing columns were assumed to be normally distributed and compared using a two-tailed paired *t*-test. *P*-values are reported as: *P* > 0.05 (ns), *P* ≤ 0.05 (*), *P* ≤ 0.01 (**), *P* ≤ 0.001 (***), *P* ≤ 0.0001 (****).

**Fig. 1:**
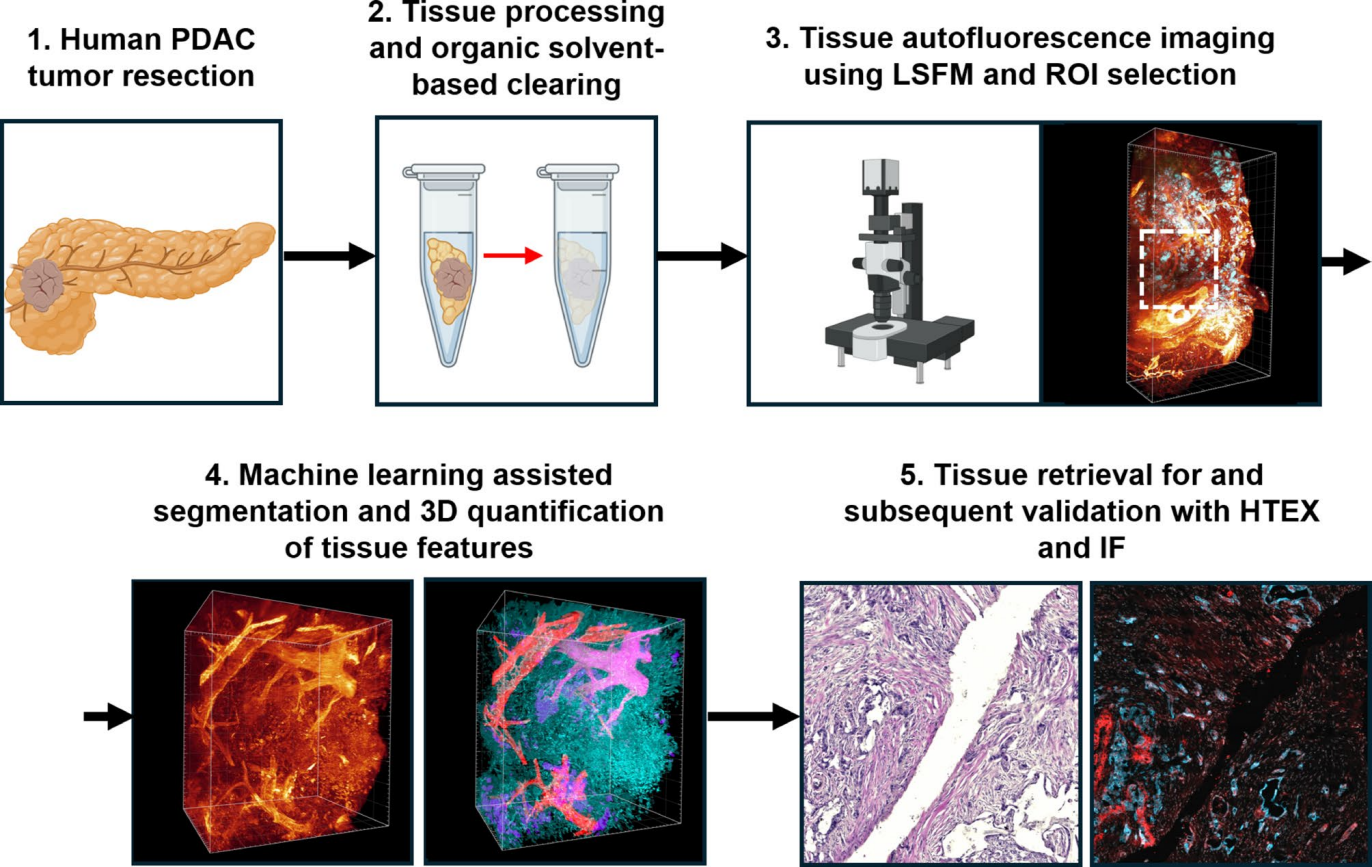
Pipeline for 3D volumetric rendering of pancreatic cancer tissue features. Acquired pancreatic cancer tissue (1) were sliced to a thickness of ~3mm and subjected to tissue preprocessing (freeze-thaw cycles and bleaching) and clearing in BABB (2) to allow for subsequent imaging by LSFM. Mosaics were generated of the entire tissue discs (3) and regions of interest containing suspected neoplasia were thereafter captured at higher resolution for segmentation and quantification (4). Notably, the method is non-destructive, and the scanned specimen can be retrieved for validation and additional analyses by HTEX and/or IF staining. Created with assets from BioRender.com.

## Results

### Processing and quantification of autofluorescent PDAC features

**Fig. 2:**
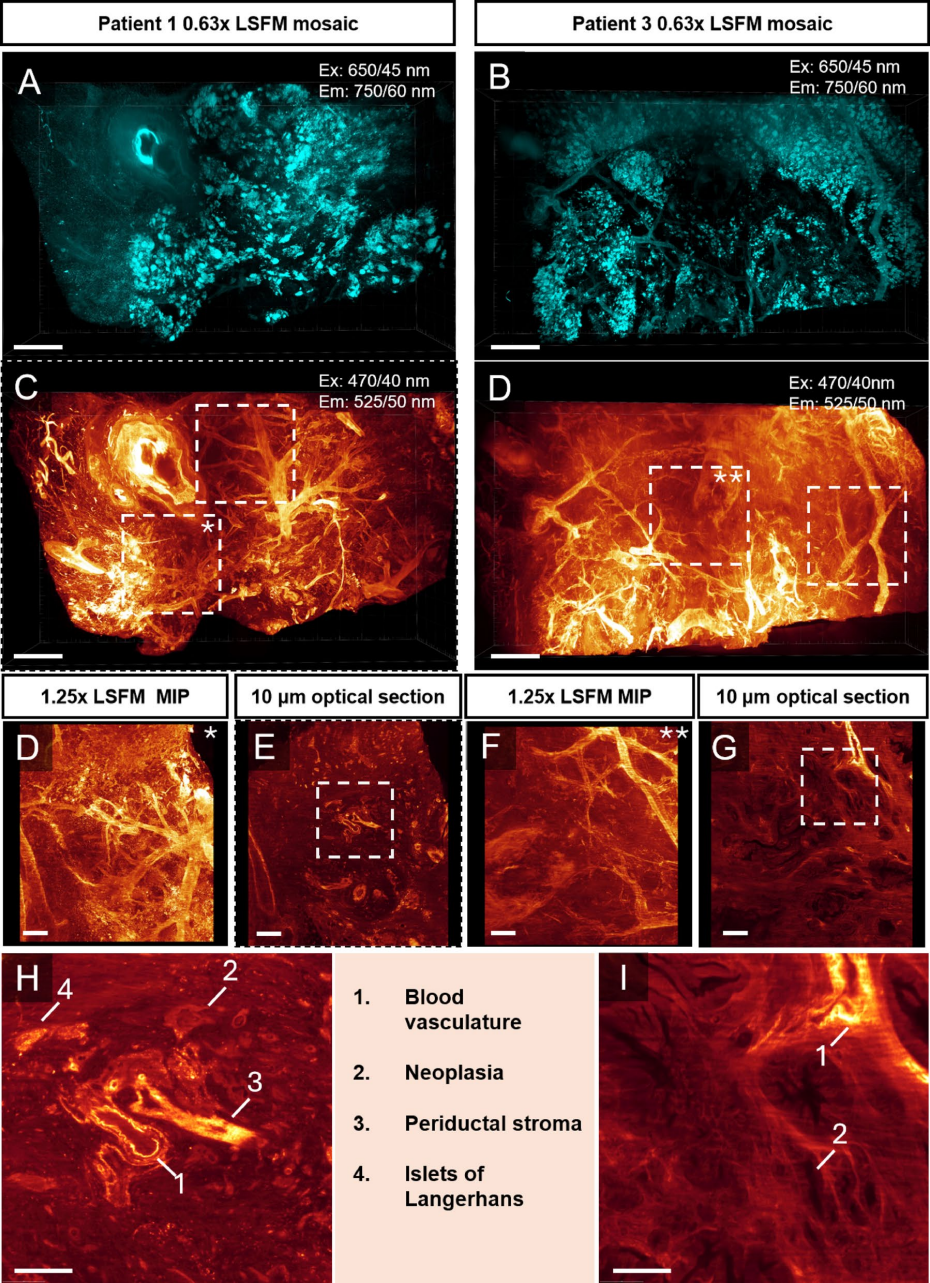
Mesoscopic identification of tissue components based on LSFM tissue autofluorescence imaging. A-D, LSFM MIP images from two patients (patient 1 and 3), scanned at a lower magnification in two separate channels; Ex: 650/45, Em: 700/60 which effectively visualizes islets of Langerhans (A-B) and Ex:470/40, Em 525/50 in which epithelium, vessels and stroma can be distinguished (C-D). E-H, Higher magnification MIP images (E, G) and optical section planes (F, H) of ROIs corresponding to broken line boxes in C and D. I, J, High magnification views corresponding to broken line boxes in (F) and (H) respectively. Optical sections in highlight the distinct AF profile that can be used to identify blood vessels, neoplastic epithelium, ductal stroma and the islet of Langerhans. The scalebar for A-D, H-G and H-I is 2 mm, 500 μm and 250 μm respectively.

To circumvent the problem of limited reagent penetration of PDAC tissues caused by its dense stroma (**Fig. S1**), we developed a pipeline (**Fig. **1) based on our previously described optical clearing protocol^1^, taking advantage of the intrinsic AF in human PDAC tissue. This allowed for segmentation of different tissue features without prior reagent or antibody labelling. The approach allows for subsequent retrieval and downstream analyses (in this case immunofluorescence and HTEX). Using LSFM, we generated 3D mosaics of cm^3^-sized tissue discs at µm resolution with scanning depths up to 4 mm, corresponding to 1000 regular 4 μm sections. Applied to primary tumour tissues from five patients that underwent surgery for PDAC and pancreas tissues from two non-cancerous donors, we hereby generated mosaic scans of PDAC tissue discs and identified regions with irregular epithelial morphology for further analysis (**Fig. **2). All discs were verified to contain PDAC tissue by a clinical histopathologist specializing in pancreas pathology. As previously demonstrated^1^, tubular structures appeared most prominent when imaged at Ex:470/40 nm, Em: 525/50 nm, including both blood vasculature and pancreatic ducts. The latter were distinguished by columnar epithelium and by periductal stroma, whereas in blood vessels, the AF profile likely correlates to elastin fibres present in arteries and veins (as exemplified in Li et al.^12^), providing visual distinction in annotation for machine-learning (ML). Further, imaging the samples at Ex: 650/45 nm, Em: 750/60 enabled visualization and quantification of islets of Langerhans, which have a distinct AF signature due to the presence of lipofuscins^1^. Although larger scale quantifications are possible at lower magnifications (See **Fig. S2**), we focused on selected regions of interest (ROI) at higher optical resolution to more effectively resolve finer structures and to reduce computational demand. ROIs were selected based on the following criteria: (1) containing suspected cancer, indicated by the epithelial morphology in LSFM optical sections, and (2) that the ROIs were not overlapping and (3) were not obscured by tissue features preventing proper laser penetration for optimal image quality and segmentation.

### Machine learning assisted segmentation and quantification of PDAC microarchitecture

**Fig. 3:**
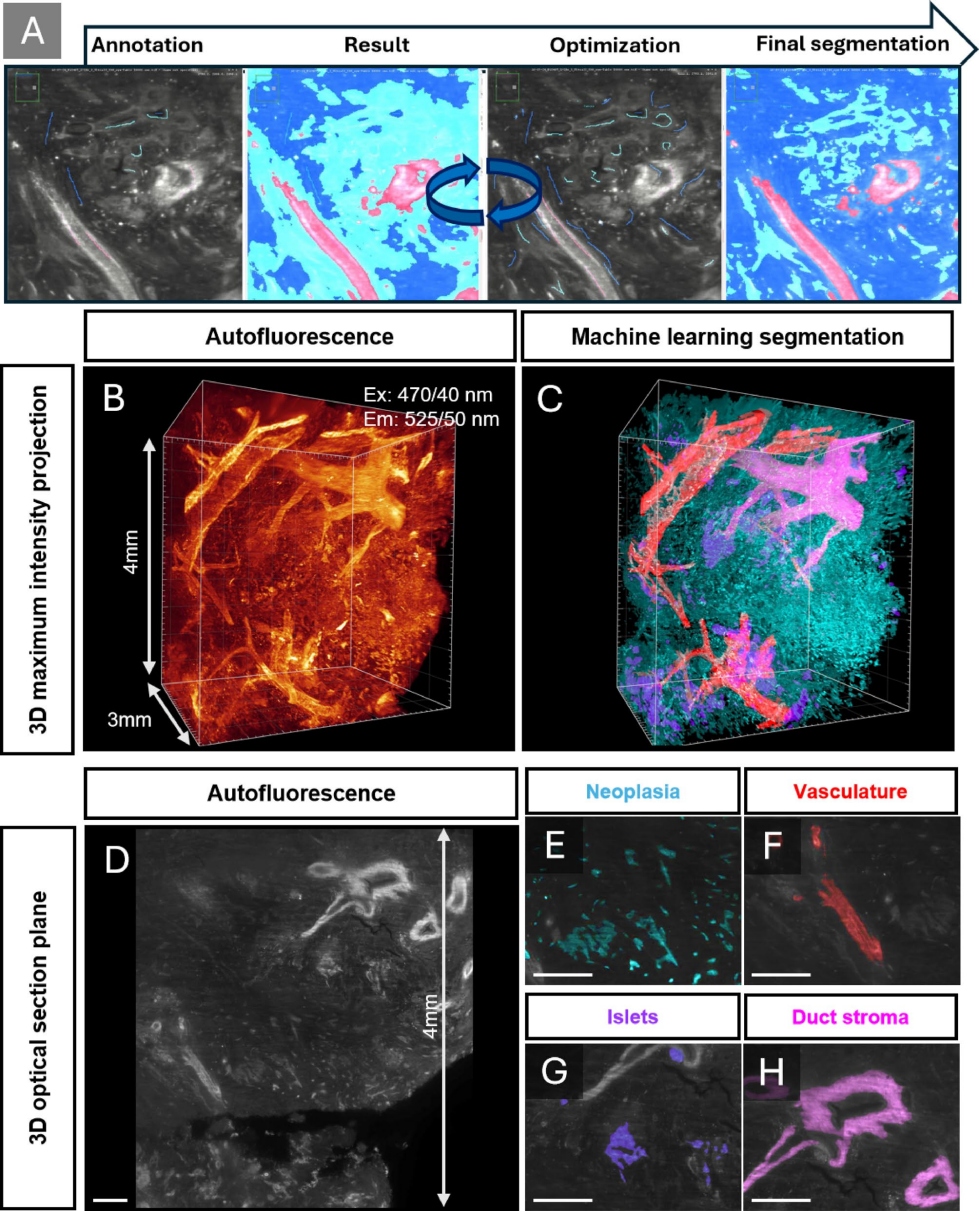
ML assisted segmentation allows for distinction and quantification of select tissue components. A, 3D machine-learning segmentation using LABKIT where the AF signal on optical sections were manually annotated into classes (neoplasia, vasculature, islets and periductal stroma) and subjected to ML which requires rounds of re-annotation and learning. B, C 3D maximum intensity projection of pancreatic cancer AF in a ROI of 4 x 4.5 x 3 mm and its corresponding segmentation. D, The AF profile on a 3D optical section of the same specimen highlighting the ability to segment features including neoplasia (E), vasculature (F), islets (G) and periductal stroma (H). The scalebar for D-H is 400 μm.

**Fig. 4:**
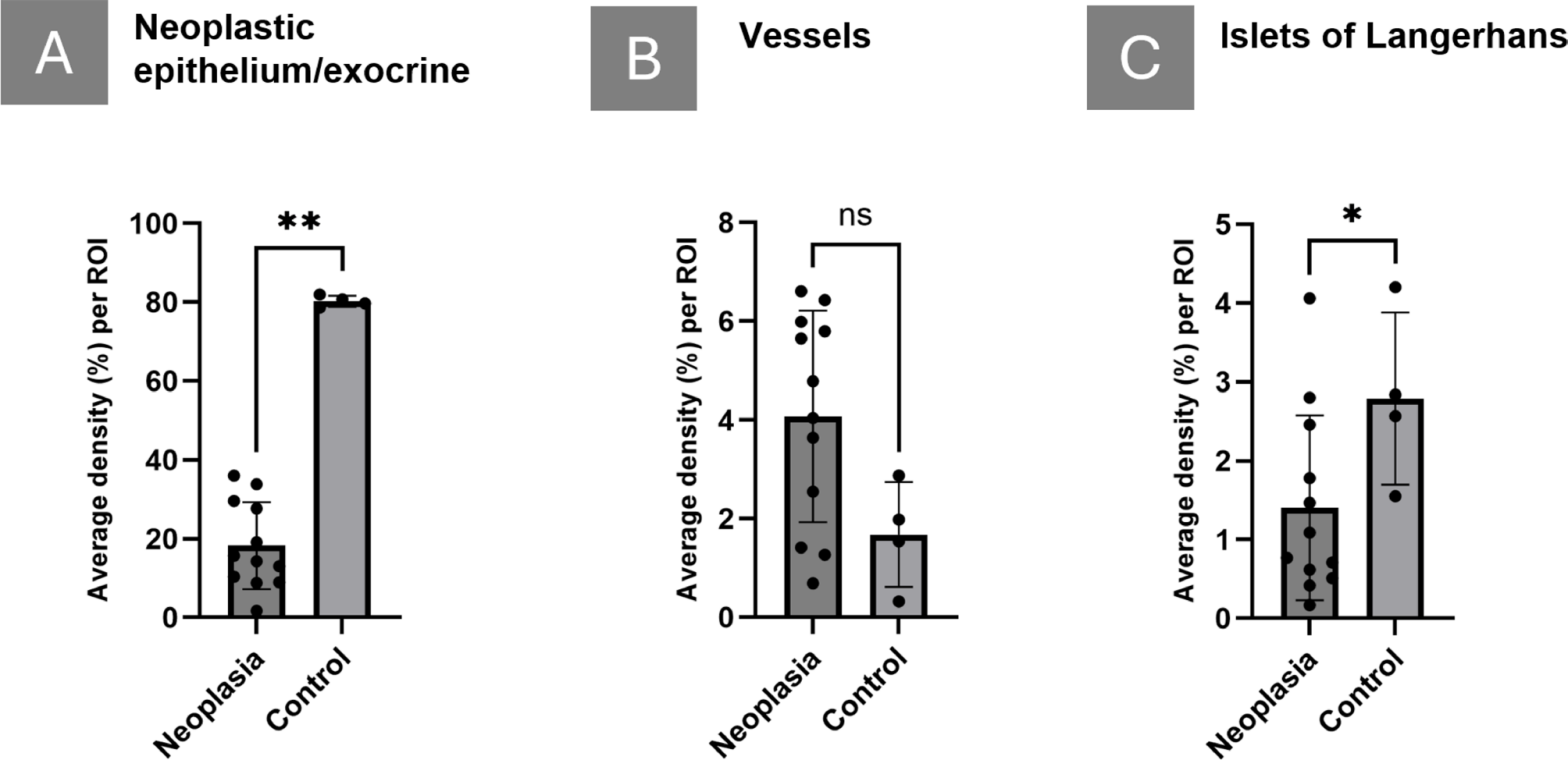
Volumetric quantification of PDAC features. Average density of neoplastic epithelium in PDAC versus healthy exocrine tissue in controls (A), blood vessels (B) and islets (C). Datapoints represent data from ROIs (see Supplemental Fig. 3) in n=5 PDAC patients and n=2 controls. Column comparison was performed using a two-tailed paired t-test. P-values are reported as: P > 0.05 (ns), P ≤ 0.05 (*), P ≤ 0.01 (**), P ≤ 0.001 (***), P ≤ 0.0001 (****). Error bars indicate standard deviation.

In total, 12 ROIs from five PDAC patients (**Supplementary Table 1**) and two ROIs from two control donors were segmented using the LABKIT extension in Imaris 3D analysis software, which employs random forest-based pixel classification. Blood vessels, cancer epithelium, islets and duct stroma were annotated and subsequently segmented through multiple rounds of learning and re-annotation until a satisfactory segmentation of the given components was achieved (see methods and **Fig. **3). As control, the pancreatic epithelium of deceased (non-cancerous) donors was segmented. As exemplified across the patient samples, this method allowed the generation of 3D volumes in Imaris for quantification in large bodies of tissue without the need for specific antibody labelling (**Fig. **3 and **S3**). After AF based segmentation, we quantified the relative volumes of each component. The average neoplastic epithelial component was found to be 18% (**Fig. **4**A** and **S4**), which is in line with previous reports regarding the relationship between cancer cells and desmoplastic stroma^13,14^. The PDAC tumour microenvironment is characterized by hypoxia and dysfunctional vasculature^15^. By optical 3D imaging, we previously noted that PDAC tissue can be highly vascularised^1^. The present high resolution LSFM data sets substantiate this observation in that vascular density on average is doubled in our PDAC tissue sample series compared to controls, accounting for 4% of the parenchymal volume (**Fig. **4**B**) although varying between and within donors. Despite the distinct fibrosis and reduced acinar density in the surgical specimens, islets were found in all PDAC ROIs. Further, although the dramatic relative reduction of exocrine tissue, the islet density in the PDAC tissue was still approximately 50% to that of healthy pancreatic control tissue (**Fig. **4**C**) where they were found primarily intermingled in the fibrotic stroma.

**Fig. 5:**
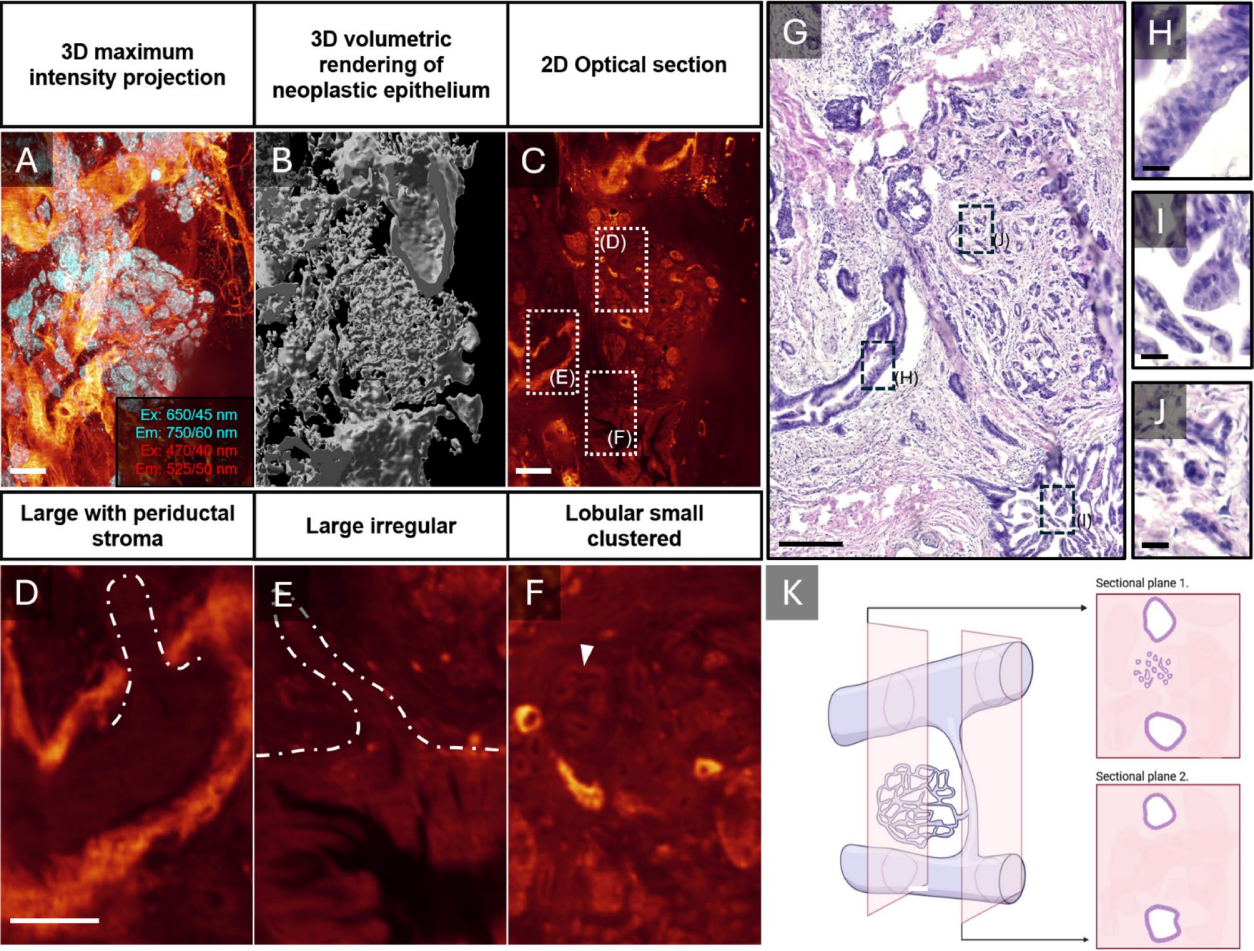
Continuous PDAC 3D structures showcase heterogeneity in morphological patterns. A-C, LSFM images displaying the morphological phenotype of a neoplastic tubular network based on AF as a MIP (A), as 3D volumes (B), and as an optical section (C). A heterogeneous morphological phenotype could be, at several instances, observed across a continuous neoplastic tubular structure. D-F, High magnification views of different morphological features corresponding to broken line boxes in (C). Observed morphologies includes small, clustered tubules intermingled with islets (D), large irregular tubes (E) and large tubes with distinct periductal stroma (F) where 2D HTEX in (G) corresponding to optical section (C) verifies the heterogeneity (H-J) observed on AF D-F. Although displaying morphological heterogeneity, the 3D segmentation (B) shows that these structures are distally interconnected (see also Supplemental Video 1), as exampled in (K) emphasizing the added value of 3D dimensionality for PDAC morphological characterization. The scalebars are 300 μm in A-C, 150 μm in D-F, 200 μm in G and 20 μm in H-J. Created with assets from BioRender.com.

The interactive and quantifiable 3D volumes of the PDAC microenvironment generated by our label free pipeline provide a more complete picture of the neoplastic tubular network. As illustrated in Fig. 5, we found several of the neoplasia’s to be continuous and interconnected. Notably, whereas the neoplastic epithelium on 2D sections often appear discontinuous, i.e., could be interpreted as isolated structures with different morphologies, the 3D data instead revealed these structures to be part of the same epithelial network spanning throughout the ROI (**Fig. **5 and **Movie S1**). In the displayed sample, two types of large ducts can be observed, one with periductal stroma (**Fig. **5**D** and **H**) and one without (**Fig. **5**E** and **I**), both interconnected by a bundle of smaller ducts (**Fig. **5**F** and **J** and exemplified in **G**). Interestingly, the neoplastic tissue was heavily intermingled with pancreatic islets, seemingly encapsulated by the stroma (**Fig. **5**A**). Together, this illustrates how the developed pipeline may facilitate 3D spatial assessments of the tumour microenvironment, including the possibility for refined classifications of tumour types/stages, that would be exceedingly challenging to perform by traditional stereological techniques.

### The developed pipeline is compatible with traditional pathological examinations

**Fig. 6:**
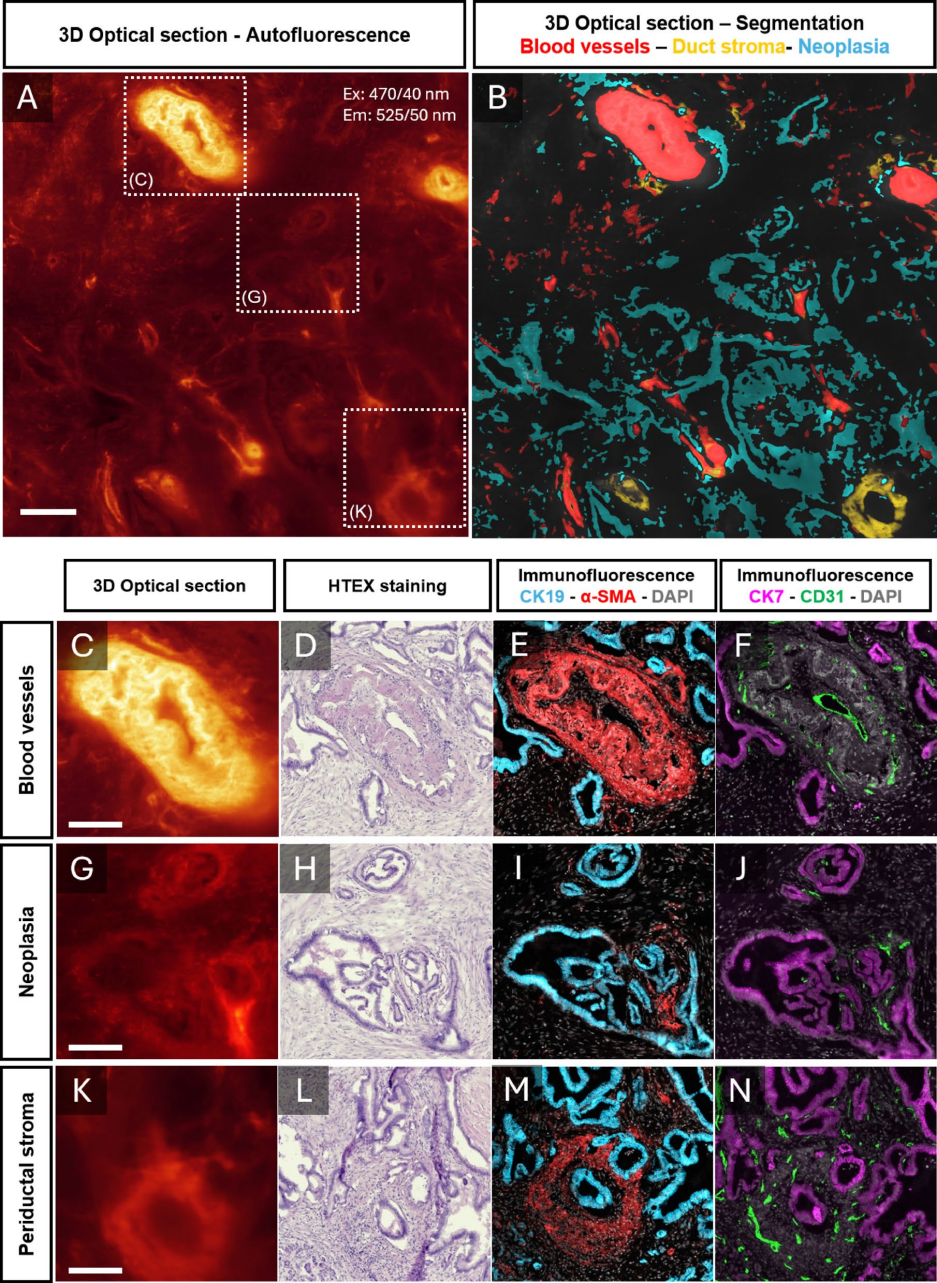
Validation of machine learning segmentation by 2D labelling. To verify segmentation of the specific AF features, the tissue discs were retrieved from clearing media, sectioned and stained for HTEX and antibodies, delineating the relevant structures. A, Representative 2D optical section of LSFM 3D scan showcasing the endogenous fluorescence. B, segmentation of blood vessels (red), periductal stroma (yellow) and neoplastic epithelium (cyan). C-N, High magnification views corresponding to broken white boxes in (A) showing assumed blood vessel (C), epithelium (G) and periductal stroma (K), stained for HTEX (D, H, L), CK19 (cyan) and $\alpha$-SMA (red) (E, I, M), CK7 (magenta) and CD31 (green) (F, J, N). These staining’s confirm the assumed identity of the segmented AF features. Scalebars are 500 μm. These authors contributed equally to this work.

To verify that the AF profile and subsequent segmentation effectively corresponded to the designated structures, the samples were retrieved from clearing media, sectioned, and processed for HTEX and immunofluorescence staining (see methods) using anti-CK19 and anti-CK7 to visualize epithelium, and anti-CD31 or anti-alpha SMA antibodies to stain blood vessels. As illustrated in **Fig. **6, machine-learning segmentation of the AF based LSFM images correlated well with the specific structures visualized by the respective antibody marker, highlighting the potential for combining the imaging pipeline (**Fig. **1) with traditional pathological assessments. Further, when comparing machine-learning assisted segmentation and immunofluorescence on matched section planes, the machine learning closely corresponded to the immunofluorescence segmentations (**Fig. S5**).

## Discussion

Antibody labelling is the most common approach to study tissue properties in 3D. This is a time-consuming approach that is highly dependent on sufficient antibody penetration, particularly in dense and thick specimens^2,9,16^. Further, the number of labelled structures is limited as per compatibility of host antibody species and spectral separation of conjugated fluorophores. Previous studies showcase the potential of generating 3D rendering of PDAC tissue features from serial sections and accompanied HTEX staining^2,17^. This methodology bypasses said issues associated with mesoscopic imaging techniques and couples well with already established deep-learning protocols for 2D HTEX. However, serial sectioning and stitching is time-consuming and associated with artefacts such as misalignment when rendering 3D images. It further requires heavy image processing to either align warped sections and/or discard poor sections which deteriorates resolution^2^. Recently, X-ray tomography was used to describe the PDAC topology in 3D^3^ combined with machine-learning assisted segmentation to classify tissue structures similar to described approach. There, samples were limited to punch biopsies of 3.5 mm in diameter in contrast to the cm wide tissue discs described in this study. Time from retrieval of a tissue sample to a viewable 3D reconstruction using the described pipeline could be achieved in a time span of 7–25 h dependent on scanning resolution and tissue size^1^, and a further 1–3 h to a finalized tissue segmentation dependent on sample complexity, affecting training time. Comparatively sized tissue scans generated using serial sectioning requires a reported 4 days of processing^2^.

We previously described how PDAC AF features can be utilized to visualize tissue characteristics of intact surgical specimens in 3D^1^. Here, we build on the intrinsic AF properties of different types of structures in tissues and show the possibility to perform segmentation and subsequent volumetric analysis in large tissue discs, using machine-learning annotation of a given tissue component. Furthermore, we highlight the possibility for downstream analysis where the processed samples can be retrieved from the clearing solution for further histological assessments, without deteriorating the specimen, as demonstrated by 2D immunofluorescence and HTEX staining’s. This compatibility is especially relevant with the rise of spatial transcriptomics and proteomics approaches, allowing high-throughput data to be generated from tissue sections with preserved spatial context^18,19^. Here, the pipeline could be used as a tool to combine layers of three-dimensional context with classification and molecular phenotyping. In the provided example, continuous structures appear heterogeneous in morphology. It is unclear whether this is due to different clonal origin, which has been described for PanINs in a study by Braxton, A. M. et al.^20^, or that the morphological variation is result of the influence of the microenvironment, where notably, stromal cells is known not only to influence intratumoral phenotype^21^, but as well shown to impact cells within individual tumour glands^22^. Together, tubular connectiveness from 3D imaging and spatial omics profiling on sections, would provide a controlled environment for further study of local factors affecting phenotypes in situ. Further, information regarding distance to blood vessels, islets or other microenvironment components could provide additional contextual depths in categorising high-throughput data.

The described pipeline can also prove useful in clinical pathology. PDAC specimens are particularly complicated and in PDAC diagnostics classifying the resection margin is challenging and highly dependent on pathology protocol and R0 definition^23,24^. Importantly, tissue handling and meticulous pathological evaluation can influence margin status beyond surgical technique. PDAC grows dispersedly, with larger distance between individual cancer cell clusters as compared to other cancer forms^25^. As such, the level of sectioning will determine the margin status in a dispersedly and haphazardly growing tumour near the resection margin. Using our pipeline, the epithelial neoplastic expansion can be mapped in large tissue volumes before sectioning for routine histopathology, allowing for selection of areas closest to the tumour margin. The feasibility of clinical use of 3D specimen evaluation for margin assessment is supported by the non-destructive protocol allowing for downstream routine H&E staining and antibody labelling. Whether it would improve margin assessment and lead to clinical impact remains to be determined. Implementation would require a systematic approach to margin assessment regions of interest followed by prospective evaluation and validation, which is beyond the scope of this paper. Further, pancreatic cancers not only display intertumoral heterogeneity (i.e. between different individuals) but also intratumoral heterogeneity (within the same tumour) which may influence resistance to therapies as well as accuracy of tumour characterisation using limited tissue sampling^26^. As exemplified, different molecular subtypes of PDAC (basal-like and classical subtypes) have been described within the same tumor^27^ as well as varying differentiation grade within a specimen, which could influence patient outcomes. This underlines the future need for characterization of larger tissue volumes to provide proper tumour classification that account for intertumoral heterogeneity.

Here, we also show the possibility to detect and quantify Langerhans islets within PDAC tissue at a mesoscopic scale. Patients with PDAC frequently have concurrent diabetes mellitus, often as an early symptom of the disease^28^. Whereas this association is well established, less is described about islet morphology and function within the pancreas in PDAC associated diabetes mellitus, these parameters, using our pipeline can now be more readily assessed in larger cohorts.

In summary, we present a pipeline for non-destructive 3D volumetric machine learning-assisted segmentation and quantification of the microarchitecture of cm^3^-sized PDAC tissues. As such, this protocol may contribute to a more precise tumour classification, while still enabling downstream high-throughput data generation and traditional 2D histopathological analyses of the same tissue specimen.

## Electronic Supplementary Material

Below is the link to the electronic supplementary material.

Supplementary Material 1

Supplementary Material 2

## Data Availability

Due to the large size of the raw and processed imaging datasets acquired by LSFM, these are available upon reasonable request to the corresponding author.
