# Supplementary material for: Rapid autofluorescence based 3D optical imaging of the pancreatic cancer milieu at mesoscopic scale – stain-free volumetric segmentation
Source: Sci Rep. 2026 May 31;16:16814. doi: 10.1038/s41598-026-54433-z (PMC13226678; doi:10.1038/s41598-026-54433-z)
Supplement: Supplementary file 2 — Supplementary Material 2 [file 41598_2026_54433_MOESM2_ESM.docx]

Supplementary materials for

**Rapid autofluorescence based 3D optical imaging of the pancreatic cancer milieu at mesoscopic scale – stain-free volumetric segmentation**

Joakim Lehrstrand et al.

Corresponding author: Oskar Franklin, [Oskar.franklin@umu.se](mailto:Oskar.franklin@umu.se)

**The PDF file includes:**

Supplemental Figure 1-5

Supplemental Table 1

Description of Additional Supplementary Files


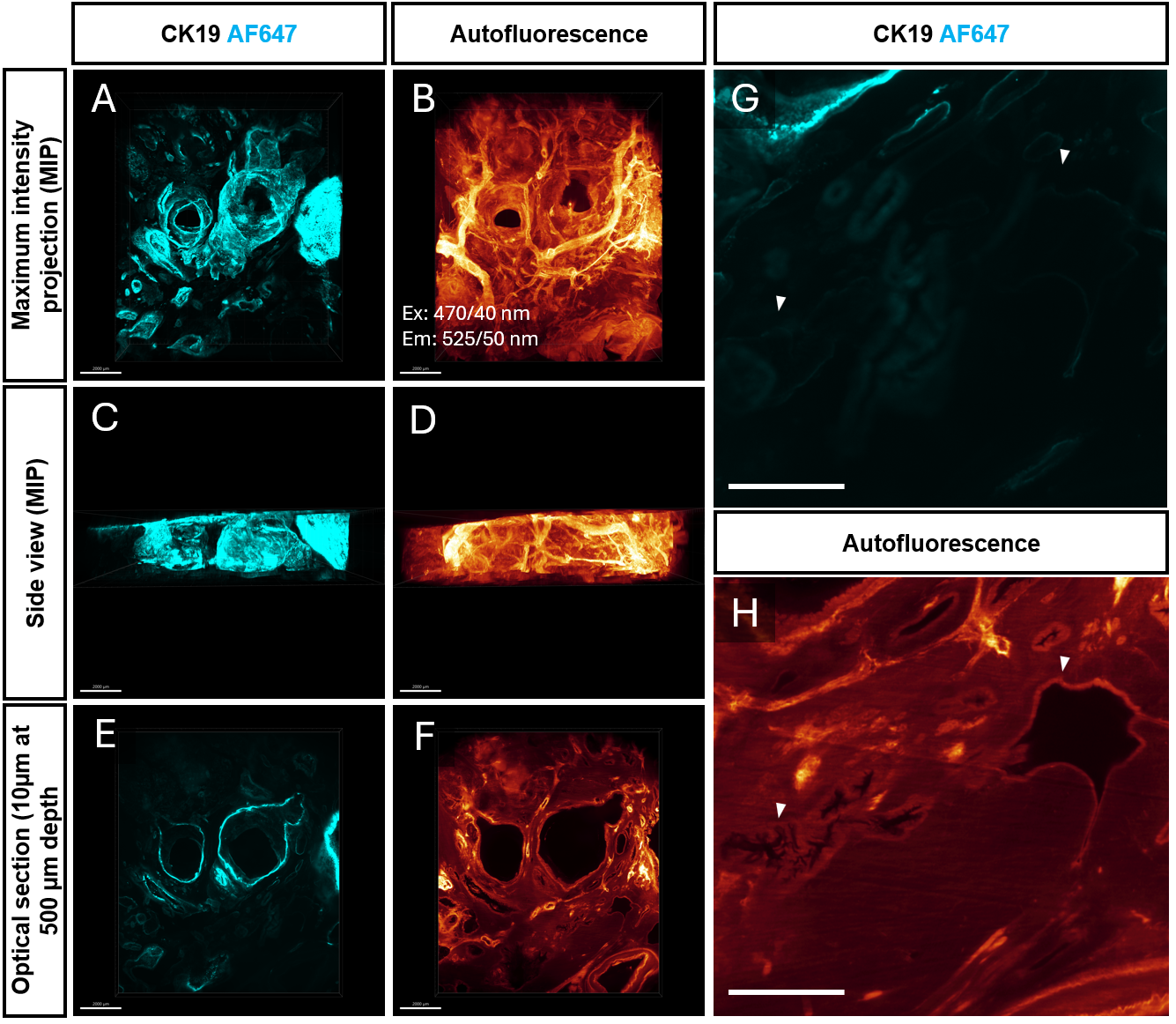
 **Supplemental figure 1**. **PDAC stroma complicates sufficient antibody penetration**. Maximum intensity projection of CK19 stained PDAC tissue slab and corresponding autofluorescent scan viewed as frontal MIP (**A**, **B**) and from the side (**C**, **D**). Insufficient antibody penetration can be observed on optical section (**E-F**) at a depth of 500 μm highlighted in G where no signal is found for epithelium easily discernible by autofluorescence in **H** (arrows in white). Scale bars in A-F are 2 mm and in **G-H** 1 mm.


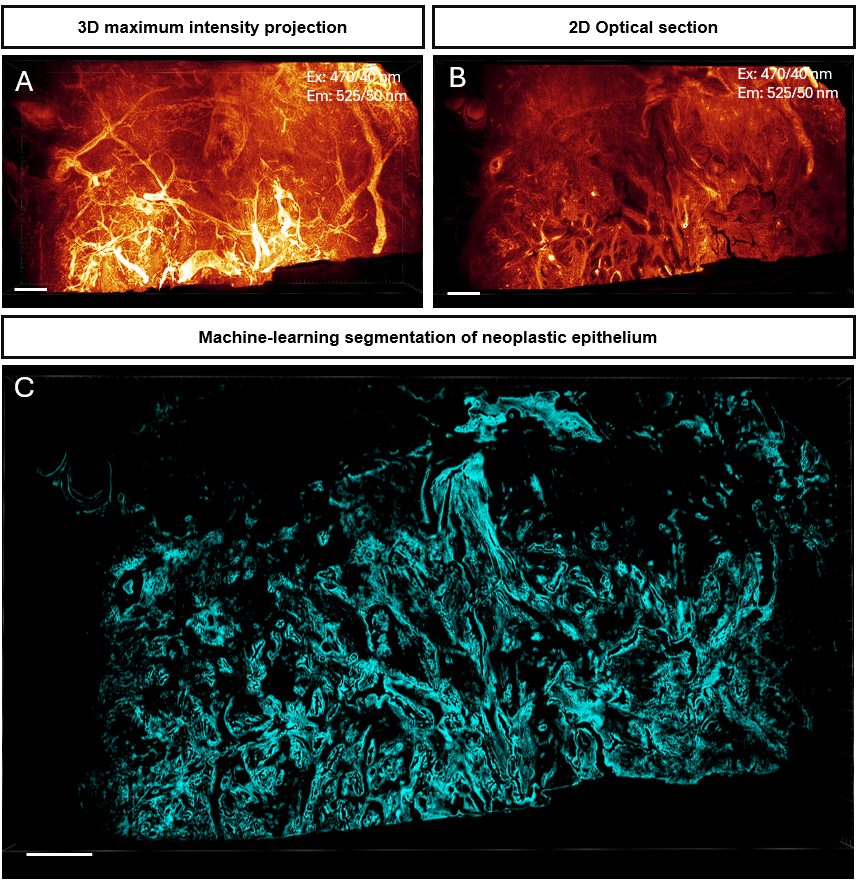
**Supplemental figure 2.** **Machine learning assisted segmentation allows large scale annotation of tissue features. A,** Maximum intensity projection image of PDAC tissue disc using LSFM at 0.63x magnification. **B**, LSFM optical section of specimen seen in (**A**). **C,** Example of machine learning annotated neoplastic epithelium based on specimen seen in (**A**, **B**). Scale bar is 1.5 mm in **A-C**.


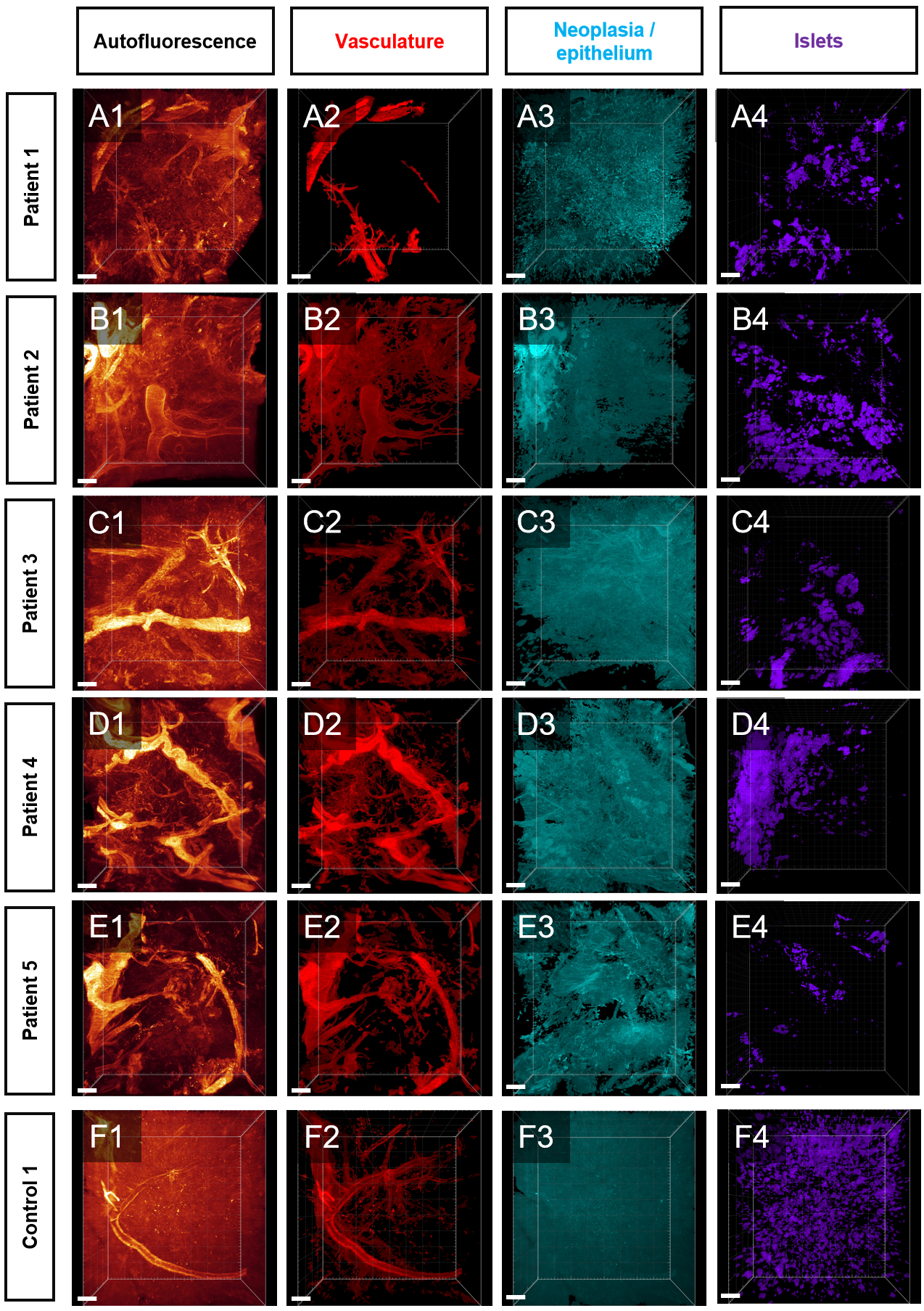


**Supplemental figure 3. Machine learning assisted 3D segmentation of PDAC microarchitecture. A-E**, Segmentation results of original AF channel (Column 1) showing representative ROIs of each patient (**A-E**) (*n*=5) and from a representative control individual (**F**). Segmentations are displayed as a maximum intensity projections with the segmentation masked over the AF in (**A**), for blood vessels (Column 2), neoplasia/epithelium (Column 3) and islets (Column 4).


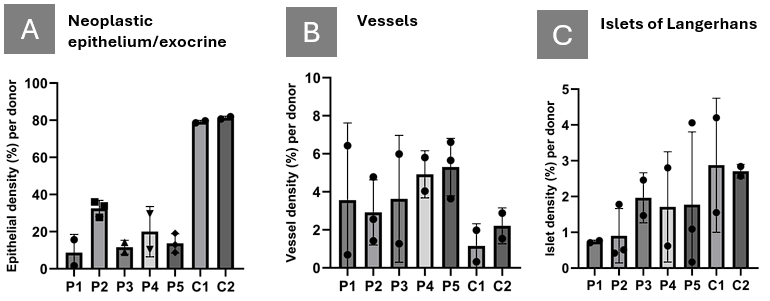


**Supplemental figure 4. Quantification of PDAC tissue features in the analyzed ROIs.** Average density of neoplastic epithelium/exocrine tissue (**A**), blood vessel (**B)** and islets (**C**) in percentage. Datapoints represent each region of interest per donor, in *n*=5 PDAC patients and *n*=2 controls. Error bars display standard deviation.

**
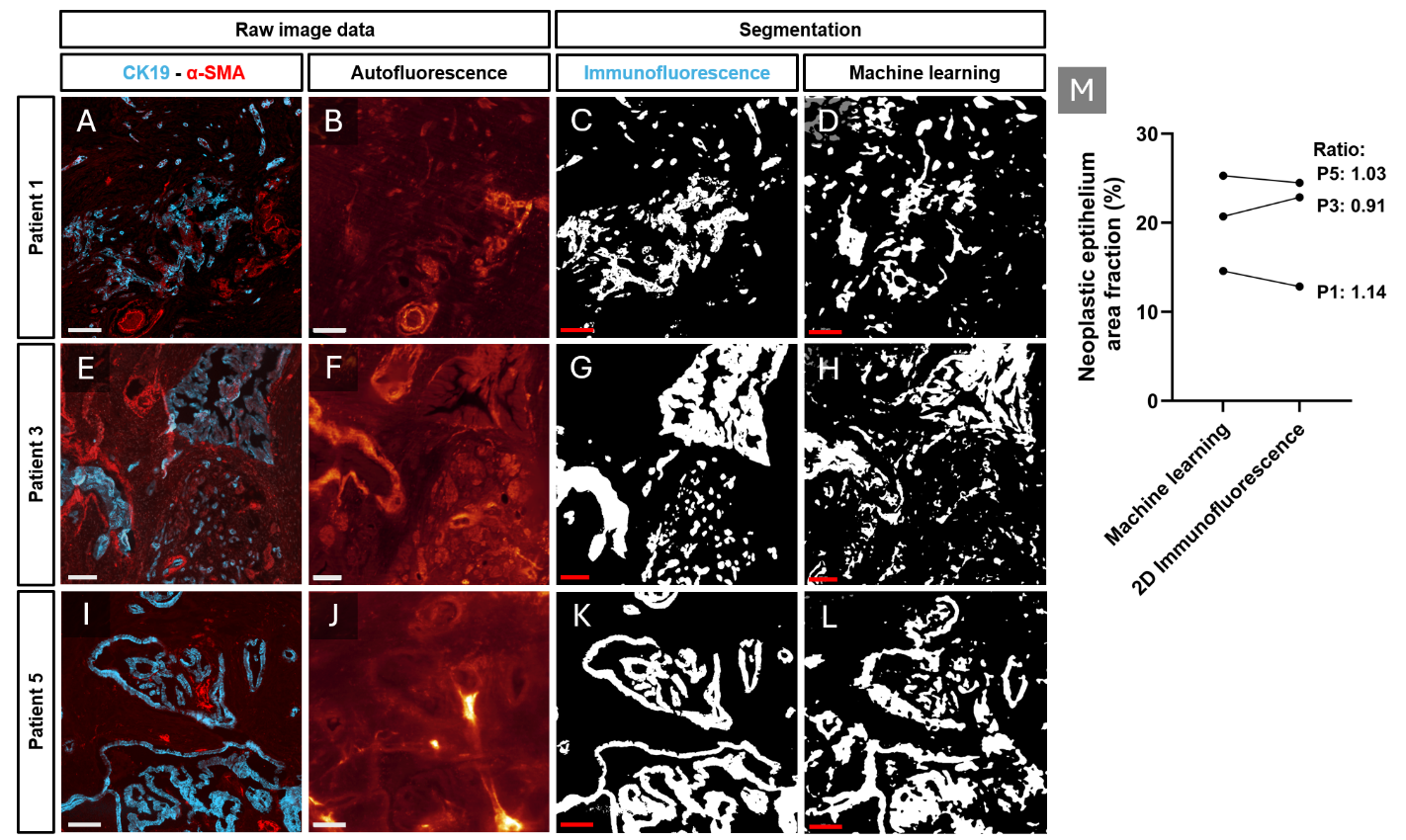
**

**Supplemental figure 5. Comparative analysis between segmentation methods.** To assess the accuracy of the machine learning assisted segmentation immunofluorescent CK19 stained sections (A, E and I) were used as reference to align a corresponding 3D optical sections plane (B, F and J) in 3 patient samples. A segmentation was performed for each CK19 staining (C, G, and K) and compared with the machine learning assisted segmentation mask in D, H and L. The area fractions were determined for each section and summarized in M showcasing the correspondence between 2D immunofluorescence data and the segmentation on 3D images with ratios (area fraction (ML): area fraction (IF)) between the two being for P1 :1,14, P3: 0.91 and P5 :1.03. Scalebars in A-L corresponds to 200µm.

**Supplemental video 1.** Example of volumetric segmentation of neoplastic epithelium (cyan) and its respective AF signal at Ex:470/40 nm, Em: 525/50 nm represented as red. Tracking the path (arrow) of a larger neoplastic duct reveals the transition into a smaller clustered branching network intermingled with islets, which might appear segregated by dense stroma if only viewed on in a 2D section.

**Supplementary table 1.**

|  | **Diagnosis** | **M/F** | **Differentiation** | **Operation** | **Tumor size (mm)** |
| --- | --- | --- | --- | --- | --- |
| **Patient 1** | **PDAC, T3N2M1** | **M** | **Low** | **Whipple** | **42** |
| **Patient 2** | **PDAC, T1N1** | **F** | **Low** | **Distal** | **19** |
| **Patient 3** | **PDAC, T2N1** | **F** | **Moderate** | **Distal** | **25** |
| **Patient 4** | **PDAC, T2N0** | **M** | **Moderate** | **Distal** | **40** |
| **Patient 5** | **PDAC, T2N0** | **F** | **High** | **Whipple** | **26** |
| **Control 1** | **Non-diabetic** | **F** | **n/a** | **n/a** | **n/a** |
| **Control 2** | **Non-diabetic** | **F** | **n/a** | **n/a** | **n/a** |
